# Method to Measure Surface Tension of Microdroplets Using Standard AFM Cantilever Tips

**DOI:** 10.1021/acs.langmuir.3c00613

**Published:** 2023-07-19

**Authors:** Pranav Sudersan, Maren Müller, Mohammad Hormozi, Shuai Li, Hans-Jürgen Butt, Michael Kappl

**Affiliations:** † Max Planck Institute for Polymer Research, Ackermannweg 10, 55128 Mainz, Germany; ‡ Technical University of Darmstadt, Hochschulstraße 8, 64289 Darmstadt, Germany

## Abstract

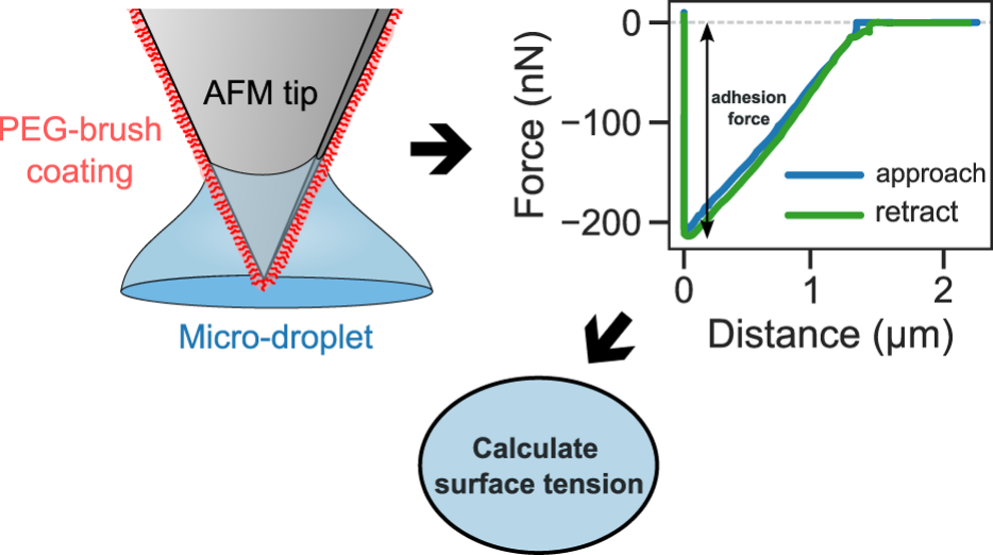

Surface tension is a physical property that is central to our understanding of wetting phenomena. One could easily measure liquid surface tension using commercially available tensiometers (e.g., Wilhelmy plate method) or by optical imaging (e.g., pendant drop method). However, such instruments are designed for bulk liquid volumes on the order of milliliters. In order to perform similar measurements on extremely small sample volumes in the range of femtoliters, atomic force microscope (AFM) is considered as a promising tool. It was previously reported that by fabricating a special “nanoneedle”-shaped cantilever probe, a Wilhelmy-like experiment can be performed with AFM. By measuring the capillary force between such special probes and a liquid surface, surface tension could be calculated. Here, we carried out measurements on microscopic droplets with AFM, but instead, using standard pyramidal cantilever tips. The cantilevers were coated with a hydrophilic polyethylene glycol-based polymer brush in a simple one-step process, which reduced its contact angle hysteresis for most liquids. Numerical simulations of a liquid drop interacting with a pyramidal or conical geometry were used to calculate surface tension from the experimentally measured force. The results on micrometer-sized drops agree well with bulk tensiometer measurement of three test liquids (mineral oil, ionic liquid, and glycerol), within a maximum error of 10%. Our method eliminates the need for specially fabricated “nanoneedle” tips, thus reducing the complexity and cost of measurement.

## Introduction

Effects of surface tension are ubiquitous in our everyday lives, whether it be the disintegration of a stream of water coming out of our shower head into smaller drops, the formation of bubbles when we use soap, or the sticking of sand particles on our wet feet during a fun beach holiday. It is a core concept in our present understanding of wetting phenomena, which emerged over the past few centuries, starting with da Vinci, in pursuit of explaining the counterintuitive rise of water inside a thin capillary tube when partially immersed vertically on its surface.^1^ Subsequent notable studies by von Segner, Young, Laplace, and Gauss formalized our current understanding of such capillary action, where surface tension was introduced as the main liquid-dependent parameter in the model. One may intuitively imagine surface tension to be a net constant tension that liquid surfaces experience in all directions, analogous to the stretched rubber membrane of a balloon. This tension is a net consequence of an in-balance in the net interaction force experienced by molecules near the liquid interface.^2^

Several measurement techniques have since been developed to measure the surface tension of macroscopic liquids. A common strategy is to use an appropriate force measurement device to directly measure the tension on liquid surfaces. Here, the Wilhelmy plate method is a classic example,^3^ where the maximum force required to pull a thin plate vertically out of the liquid surface is measured. Surface tension can then be obtained by dividing the measured force by the wetted contact perimeter of the plate. On the other hand, methods such as the pendant drop method,^4^ spinning drop method,^5^ or oscillating drop method^6^ rely on optical observations of the liquid drop shape under specific conditions to evaluate surface tension by solving the Young–Laplace equation or Rayleigh’s equation.^7^

While surface tension measurement of bulk liquids is simple using commercially available instruments based on the above techniques, they are, however, not suitable for microscopic measurements, where the available liquid sample volume is extremely low in the range of micrometer-sized droplets. Such small-scale measurements can be especially useful to improve our understanding of some important natural phenomena, such as how atmospheric aerosols impact climate change processes and human health^8^ or the nature of tiny secretions in the legs of certain insects which enable them to stick to most surfaces.^9^ One of the first attempts in making such a measurement was by performing a Wilhelmy-like experiment using an atomic force microscope (AFM). McGuiggan and Wallace^10^ attached a cylindrical quartz rod of roughly 100 μm to a tipless AFM cantilever probe, which was used to measure the liquid adhesion force and calculate the surface tension. While their method gave reasonable values for low surface tension liquids such as tetradecane, the method, however, underestimated the values for water by 44% when compared with macroscopic results. This discrepancy was attributed to imperfections in the rod shape and water contamination. An improvement to the above method was reported by Yazdanpanah et al.,^11^ where a “nanoneedle” of gallium–silver alloy was grown on the sharp tip at the end of a standard AFM cantilever. These nanoneedles (diameter ∼100 nm) have a more well-defined cylindrical geometry, which allowed for precise surface tension measurement of liquids, including water. A similar method using colloidal probe AFM has also been demonstrated for a capillary-condensed liquid bridge formed between the spherical probe and the substrate.^12,13^ An alternative approach was to track the droplet oscillations induced by either coalescence using optical tweezers^14^ or while under flight when ejected through an inkjet nozzle.^15–17^ The droplet oscillations, whose resonance frequency modes depend on the liquid surface tension based on Rayleigh’s theory,^7^ can then be analyzed using high-speed optical detectors. Similar experiments could also be performed on hemispheric sessile drops using an AFM by measuring the oscillations when a liquid interface comes in contact with a hydrophobic colloidal probe.^18^

Based on the above review, AFM provides in principle a relatively easier way to measure the surface tension of small droplets without the need to construct specific experimental setups. Further, AFM is quite versatile since even submicrometer-sized droplets can be probed with high resolution, which is not possible using alternative optical methods. Presently, the “nanoneedle” tip-based method^19^ shows the most promise, having been used by several groups to study the surface tension of aerosol droplets, for example.^8,20^ However, a clear drawback of this method is the need to fabricate such nanoneedles precisely on the cantilever tip, which are not easy to prepare and would be expensive as a commercial product. Is there a way to circumvent the reliance toward such special tips and instead make similar measurements with standard pyramid-shaped tips that are widely used for general AFM imaging?

Although it is possible to obtain AFM force–distance curves when a pyramid-shaped tip makes contact with a liquid droplet, there are, however, several challenges to calculate the desired surface tension value, which is hidden within the measured force data. First, the theoretical capillary force interaction between a liquid drop and a pyramid shape can be precisely estimated only by numerical simulations, making the analysis procedure complicated.^21^ Second, the surface properties and precise shape of the AFM tip significantly influence the measured force values. Small structural or chemical heterogeneities on the tip surface can lead to pinning of the liquid contact line, resulting in a different force response. In such cases, precise knowledge of the liquid contact angle with the tip or point of contact line pinning is essential to make reliable numerical predictions. Fabié et al.^22^ reported that AFM force curves show a good agreement with simulations if the liquid contact line is assumed to remain pinned to the facets of a hydrophilic AFM tip at some fixed point. However, for hydrophobic coated tips, the liquid contact line recedes at a certain contact angle and also undergoes pinning at multiple intermediate points when the tip is retracted away from the liquid. This complex dewetting process could not be precisely modeled, and thus, it was not possible to obtain simulated force curves that follow the experimental curves. Hydrophilic tips may thus, in principle, be used to back-calculate the surface tension of microdroplets by fitting the obtained AFM force curves to simulation data. However, an additional problem of such tips would be the continuous loss of drop volume whenever they make contact, which is an undesired side effect of the contact line pinning as a result of large contact angle hysteresis that hydrophilic surfaces typically show. This continuous contamination of the hydrophilic tip during the measurement process further complicates the analysis of force curves.

In order to enable the use of standard pyramidal AFM tips for surface tension measurements, it is essential to modify the tip surface such that it has a low contact angle hysteresis with any liquid that should be probed, i.e., to render the tips amphiphobic. This could, in principle, minimize any local pinning events or sticking of liquids when they are in contact with the tip during the measurement, and thus, one could obtain force curves which closely follow the ideal theoretical scenario. While hydrophobic coating with a fluoropolymer, for example, does reduce the hysteresis to a certain degree, they have poor antiwetting properties to low surface tension liquids. Two common strategies to make surfaces amphiphobic is by coating them either with a nanostructured^23^ or a lubricant^24^ layer. Both of these methods, however, would not be suitable for our needs since such amphiphobic layers are usually several micrometers thick, which could change the tip shape or could contaminate our sample liquids if we choose the lubricant-based coating method. Recently, it has been shown that the polymer brush coating can be used to obtain surfaces with very low contact angle hysteresis (less than 5°) due to the high chemical and physical homogeneity of the dense brush layer,^25,26^ which behaves similar to a thin lubricating liquid-like film. These polymer brushes have a nanometer-scale coating thickness, making them an ideal candidate for modifying the tip surface.

In this work, we present a method to perform equilibrium surface tension measurement on microdroplets with an AFM using standard pyramidal tips coated with a polymer brush. A simple one-step process was adapted from a previously reported study^27^ to obtain a hydrophilic polymer brush coating on the tip surface. The low contact angle hysteresis of the coated tip prevented liquid drops from sticking to the AFM tip, despite being hydrophilic. Further, we used the Surface Evolver software^28^ to numerically simulate the configuration of a liquid drop sitting on a flat surface and interacting with a pyramid or a cone-shaped tip from the above.^21,22^ The simulated force–distance curves were compared with the experimental AFM data to calculate the surface tension. Our method attempts to simplify microscale surface tension measurements and also help progress scientific understanding of the various processes governed by wetting phenomena on small scales.

## Methods

### Simulation Scheme

**Figure 1:**
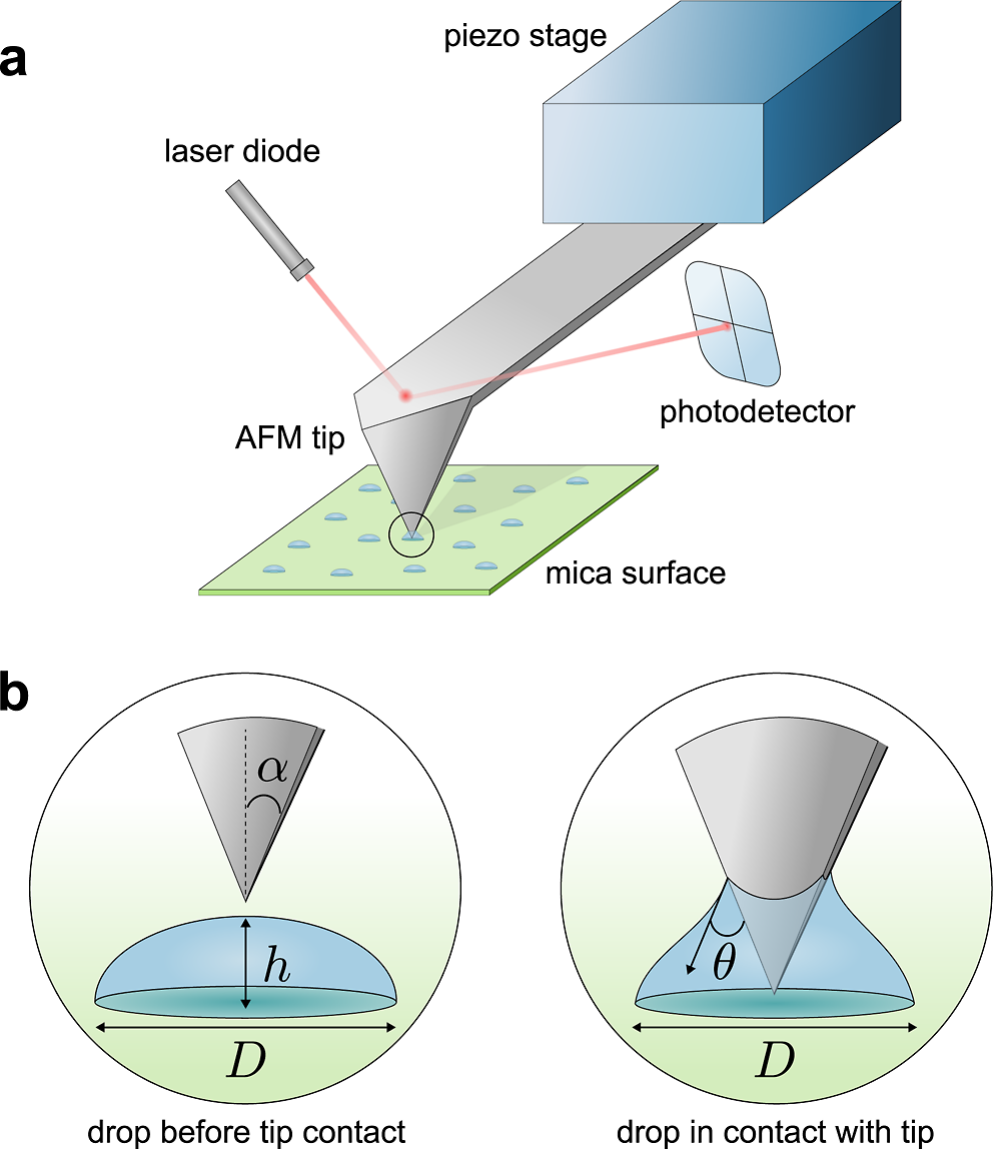
(a) Schematic of an AFM experiment on microdroplets deposited on a mica surface. (b) Magnified view showing the interaction process of the AFM tip with the liquid drop (circled region in a). Initially, a liquid drop is pinned to the surface with contact diameter, *D*, and height, *h*. During force measurement, the drop makes a contact angle, θ, with the tip surface. The tip shown here has a regular square-pyramidal geometry of half-angle, α.

The condition of a sessile liquid drop in contact with an AFM tip was simulated using Surface Evolver software.^28^ The AFM tip was modeled to be either of a regular square pyramid or a cone geometry, with a half-angle, α. The drop having a volume *V* of liquid with surface tension γ was assumed to remain pinned to the bottom surface, following a fixed circular contact line with diameter *D*. The apex of the pyramid/cone tip was in contact with the substrate below at the center of the drop. On the top, the drop had a constant contact angle, θ, with the AFM tip surface. The contact angle boundary condition was incorporated into the model by calculating the energy difference between the tip–liquid and tip–air interfaces using the Young–Dupré equation: γ_tip-air_ – γ_tip-liquid_ = γ cos θ. Here, an appropriate line integral along the tip–liquid contact line was defined for the tip geometry in order to correctly calculate the interfacial energy over the tip–liquid contact area.^28^ Effects of gravity can safely be neglected, as droplet sizes are far below the capillary length. All lengths and forces involved were normalized w.r.t. *h* and γ*h*, respectively, where *h* is the undisturbed height of the sessile drop without making contact with the tip (Figure 1b). Gravity has an insignificant effect on the microdroplet shape here since the Bond number is very low. Hence, the sessile drop assumes the shape of a spherical cap, and the drop volume relates to *h* through the analytic expression,^29^*V* = π*h*(3*D*^2^/4 + *h*^2^)/6, which simplifies the volume normalization. An appropriate solution routine was written in the software to “evolve” the shape of the liquid drop, starting from a polyhedron initial condition to its final equilibrium state by following successive mesh refinement and surface energy minimization steps (script files available in the public GitHub repository https://github.com/PranavSudersan/afm_pyramid). In this case, the net vertical adhesion force, *F*_adh_, between the tip and the liquid drop is given by
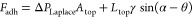
Here, Δ*P*_Laplace_ is the Laplace pressure difference, *A*_top_ is the contact area between the tip and the drop projected on the horizontal plane, and *L*_top_ is the perimeter of the contact line between the tip and the drop. Note that the van der Waals adhesion force between the tip and the substrate are negligible relative to the capillary force and hence not considered. For comparison with simulation data, the above equation needs to be rewritten in the normalized inverted form
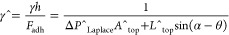
Here, Δ*P̂^*_Laplace_ = Δ*P*_Laplace_*h*/γ, *Â*_top_ = *A*_top_/*h*^2^, and *L̂*_top_ = *L*_top_/*h* are the output parameters from the simulation, which were used to calculate the liquid surface tension, expressed in nondimensional form as γ̂ = γ*h*/*F*_adh_. The simulations were iteratively repeated for a range of *D̂* = *D*/*h*, θ, and α values for both pyramid and cone geometries to obtain a map of nondimensional surface tension values for a specific set of parameters. The contact angle, θ, was taken from the experimentally measured values between the test liquid and a flat substrate of surface chemistry identical to that of the AFM tip. Since the tip is hydrophilic, θ was typically less than 50° (see the Supporting Information, Figure S5). *F*_adh_, *D*, and *h* can be easily obtained by experimental AFM measurements, while α can be measured by scanning electron microscopy (SEM), as will be described in the subsequent sections. In this manner, the experimentally measured parameters can be utilized to calculate the liquid surface tension from the simulated γ̂ values.

### Wilhelmy Plate Method

Surface tension measurements were carried out on three test liquids: mineral oil (RTM-13, 75 cSt, Paragon Scientific Ltd., UK), glycerol, and ionic liquid (trihexyltetradecylphosphonium bis(trifluoromethylsulfonyl)imide, >98%, IOLITEC GmbH, Germany). For bulk liquid measurements, we followed the Wilhelmy plate method using a commercial tensiometer (DCAT 11EC, DataPhysics Instruments GmbH, Germany) and a platinum–iridium plate of width 19.9 mm and thickness 0.2 mm. Before attaching the plate to the tensiometer’s force sensor, it was rinsed in ethanol and subsequently burned with a butane torch until it glows red, thus removing any contaminants. 10 mL of test liquid was pipetted into a Petri dish placed under the plate. The instrument’s software control was used to bring the plate close to the liquid surface. The plate was then partially dipped into and out of the liquid with a constant vertical speed of 0.1 mm/s while simultaneously recording the detected force values. The maximum force measured while the plate is retracted out of the liquid was used together with the plate geometry values to calculate surface tension.

### Cantilever Coating

The cantilever tip model RFESPA-75 (spring constant of ∼3 N/m, Bruker) was used for all AFM measurements. Polyethylene glycol (PEG) chains were grafted on the cantilever tips by following the silanization method reported by Cha et al.^27^ The cantilevers were first cleaned in an oxygen plasma chamber (Diener Electronic Femto) for 2 min at 48 W power and then subsequently placed in a solution mixture comprising 2 μL of 2-[methoxy(polyethyleneoxy)propyl] trimethoxysilane (90%, 6–9 PEG units, abcr GmbH, Germany), 8 μL of hydrochloric acid (fuming, ≥37% assay, Sigma-Aldrich), and 10 mL of toluene (≥99.8%, Fischer Scientific, UK). After 18 h, the cantilevers were cleaned in an ethanol bath for 10 min to finally obtain PEG-brush-coated hydrophilic cantilever tips. AFM experiments with the cantilever were subsequently performed within a few hours post-coating. An identical cleaning and coating procedure was also performed on a flat silicon wafer. Dynamic contact angles (DataPhysics OCA 35 goniometer) of glycerol, mineral oil, and ionic liquid were subsequently measured on the resultant PEG-brush-coated silicon wafer by observing a 10 μL drop slide over the wafer tilted by 10°. The measured receding contact angle values were used for the surface tension calculation by the AFM method, as described later.

### Droplet Generation

Small microdroplets were deposited on a freshly cleaved mica surface for AFM measurements. The droplet deposition was carried out with the help of a micropillar array of polydimethylsiloxane (PDMS) fabricated by a soft lithography procedure reported by Greiner et al.^30^ In brief, an array of micropillars (diameter of 5 μm) was first prepared by curing a thin layer of SU8 photoresist under UV, which was used as the master template to subsequently prepare the PDMS pillar array in a two-step molding process. The resultant PDMS array was then smeared with the test liquid, which was used to stamp small droplets on the mica surface. Drops with a size range of 5–25 μm contact diameter were obtained by following this method.

### AFM Measurements

**Figure 2:**
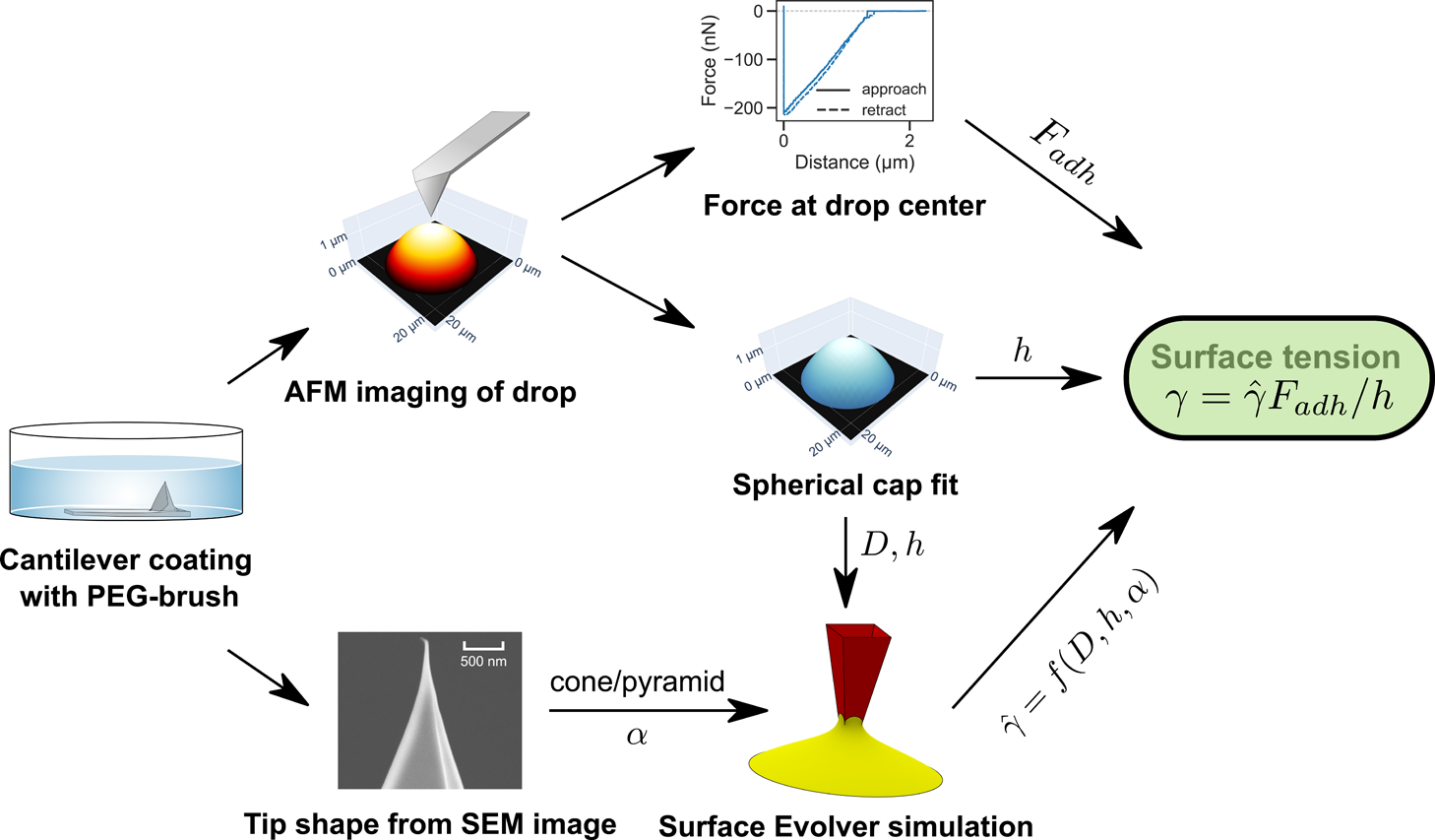
Flowchart summarizes the procedure to measure surface tension. See the text for detailed description.

Measurements on microdroplet liquids were performed using the JPK NanoWizard 3 AFM (Bruker) for mineral oil, glycerol, and ionic liquid. A custom-made rubber gasket was fitted between the cantilever holder and mica surface during measurements to maintain the microdroplets in a sealed environment and minimize evaporation. After loading the PEG-brush-coated cantilever and the droplet carrying mica surface onto the AFM sample stage, the system was left for 60 min to allow the droplets to equilibrate with the surrounding sealed chamber. Following the equilibration step, the droplets were imaged under the intermittent contact mode (tapping mode). First, the cantilever tip was positioned close to a drop of interest with the aid of the AFM’s built-in optical microscope. The cantilever deflection sensitivity and spring constant were then calibrated using the contact-free thermal noise calibration method,^31^ available within the AFM software. The cantilever was tuned to a driving frequency slightly below its resonance frequency with a specific target oscillation amplitude. Images were taken under high feedback gain and soft tapping conditions, and an appropriate scan line rate and area size were chosen to obtain a good overlap between the height trace and retrace curves. For example, droplet height images for glycerol were captured at a scan line rate of 0.7 Hz and a scan area size of 22 × 22 μm with the cantilever tuned at a target amplitude of 40 nm (Figure 2). Since the droplet sizes were comparable to the scan area, high image resolution was unnecessary for analysis. All images were thus recorded at 128 × 128 pixel size to speed up the scanning process. Using the captured drop AFM image as a reference, the cantilever was precisely positioned to the center of the drop by software control of the AFM’s piezo motion stages and force spectroscopy measurements were done. Here, the cantilever tip approached and penetrated the drop, made contact with the mica surface, and finally retracted back vertically out of the drop (Figure 1). The approach/retract speed was 0.1 μm/s. The approach/retract distance was set depending on the height of the drop obtained from the previously captured drop image. The force trigger set point was set to 10 nN in order to detect a hard contact between the tip and the mica surface, which would then initiate the retraction cycle of the force curve. Force curves were recorded at 1000 Hz sample rate and repeated three times for each drop. After force measurements, the same scan area was imaged again to check for any possible loss of drop volume as a result of liquid contact with the tip or due to evaporation. Measurements were repeated with two independently coated cantilevers each for mineral oil, glycerol, and ionic liquid, i.e., six cantilevers in total. For each cantilever, images and force curves of eight drops were recorded. Thus, 16 drops were measured for every liquid.

### Tip Shape Estimation

**Figure 3:**
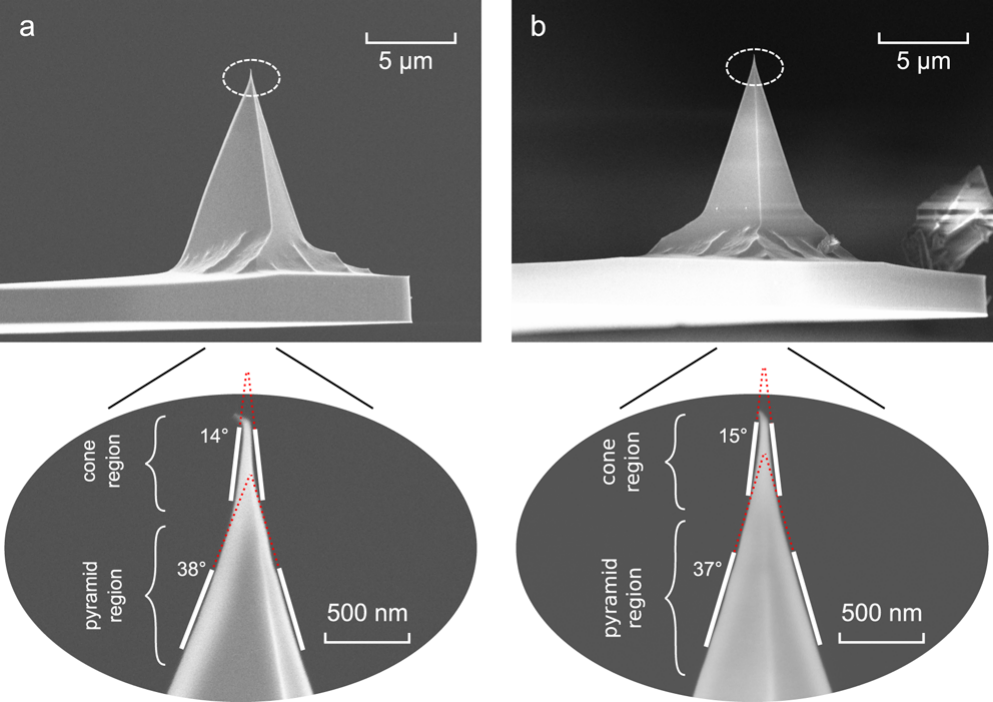
SEM of cantilever tips (model: RFESPA-75) imaged from (a) side view and (b) front view. The insets show magnified images close to the tip apex (marked by the dashed ellipse), indicating a “cone” or “pyramid”-like tip geometry. The corresponding tip full-angles for each shape are marked (see the text for details).

Since commercially available cantilever tips typically have tapered ends for better sharpness of the tip, the precise tip shape within the region making contact with the microdroplet needed to be determined. Tips were imaged after droplet experiments from both front and side views using a LEO Gemini 1530 Scanning Electron Microscope (1 nm point resolution, Zeiss, Germany). The cantilevers were mounted on the SEM sample holder with the help of carbon tape before imaging. The shape of the tip within 1 μm length scales close to the tip apex was specifically focused on. The tip full-angle was obtained by drawing straight lines following its two extreme lateral edges and measuring the angle between them using Inkscape graphics editor (Figure 3). For our analysis, we work with tip half-angle α which is the full-angle value divided by 2. The angle measured can differ depending on the region of the tip being considered since the tip deviates from its ideal regular pyramid shape and gets sharper close to its apex. In order to simplify analysis, the tip shape was classified into two regions: (1) cone-shaped very close to the apex and (2) pyramid-shaped far from the apex. A pair of lines was manually fitted on the edges for these two regions to obtain the corresponding tip full-angle. The above process was repeated for both front and side view images of the tip, and the obtained tip full-angles were averaged for each shape and used to obtain the half-angle α. For the case of pyramid however, the tip full-angles measured this way correspond to the angle, α_opp_, between its opposite lateral edges due to its orientation in the SEM images. Since our model defined the half-angle of the pyramid to lie between its adjacent lateral edges, we use the geometric relation  to obtain the true value of α from α_opp_ measured via SEM. For RFESPA cantilever tips, the average cone half-angle was ≈7°, and the average pyramid half-angle was ≈13° (obtained from the above relation). Since our test liquid drop heights lie between the cone and pyramid regions, the exact tip shape to be considered for surface tension calculations can be ambiguous. Thus, here, we consider both pyramid and cone geometries independently for further analysis.

### Surface Tension Calculation

AFM measurements of drop shape and force–distance curves combined with the knowledge of tip shape provide all the necessary ingredients to estimate the surface tension of the liquid drop. Here, we describe the general calculation procedure of combining experimental and simulation data to obtain surface tension (Figure 2): the above procedure was automated in Python to directly calculate surface tension from the raw output data of JPK NanoWizard 3 AFM. Here, the “measuredHeight” and “vDeflection” channels of the data were used to obtain the resultant drop image and force–distance curve. The scripts used for the analysis are available in the public GitHub repository (https://github.com/PranavSudersan/afm_surface_tension).

1. The AFM height image data of each drop was fitted with a spherical cap shape. The fitted cap parameters were used to obtain the contact diameter, *D*, drop height, *h*, and volume, *V*.
2. The adhesion force, *F*_adh_, was obtained from the minima of the retraction cycle of the force–distance curve which was measured for the corresponding drop.
3. The tip shape was considered to be both a pyramid and a cone. The corresponding tip half-angle, α, was taken to be half of the measured average tip full-angle obtained via SEM.
4. The normalized surface tension, γ̂, was obtained from the simulation data using experimentally measured *D̂* = *D*/*h* and α values. Here, the contact angle, θ, between the liquid and the tip was assumed based on the macroscopic experimental receding contact angle measurement of the liquid with a flat PEG-brush-coated silicon wafer substrate.
5. Finally, the surface tension was calculated in real units from the above γ̂ value together with the experimentally measured *F*_adh_ and *h* values using the relation γ = γ̂*F*_adh_/*h*.

## Results and Discussion

The precise nature of the tip shape to be considered for calculation is ambiguous because the “cone” and “pyramid” regions of the tip roughly transition at the same length scale as the drop height (≈1 μm). Thus, the calculations were performed for both geometries. We see that the pyramid approximation gives a better estimate of surface tension for mineral oil and ionic liquid relative to their macroscopic values (<5% error). For glycerol, the cone and pyramid approximation, respectively, over- and underestimates the surface tension within a 9% error relative to the tensiometer measurements (Figure 6 and Table 1).

**Table 1:** Summarized Results Showing the Range of Drop Contact Diameter, *D*, and Drop Volume, *V*, Measured from AFM Images^a^

liquid	*D* (μm)	*h* (μm)	*V* (fL)	θ (deg)	γ_AFM_ (mN/m)		γ_Wilhelmy_ (mN/m)
	[min–max]	[min–max]	[min–max]		cone	pyramid	
mineral oil	9–17	0.7–1.4	30–132	10	30.3 ± 3.6	28.4 ± 1.3	28.4 ± 0.1
ionic liquid	10–20	0.7–1.9	30–307	10	38.7 ± 0.6	31.5 ± 0.5	30.1 ± 0.1
glycerol	5–25	0.3–2.7	3–331	40	74.5 ± 4.4	62.9 ± 4.6	59.5 ± 0.2

a The assumed tip–liquid contact angle, θ, was used to calculate surface tension (γ_AFM_) from AFM data for each tip shape, which are compared to macroscopic measurements (γ_Wilhelmy_).

**Figure 4:**
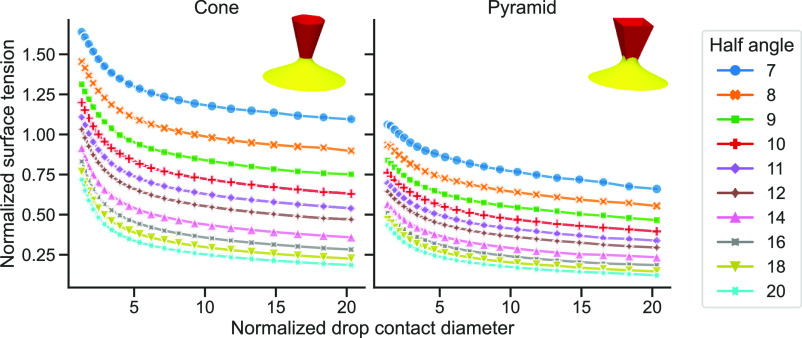
Simulation curves showing normalized surface tension, γ̂ = γ*h*/*F*_adh_, as a function of normalized drop contact diameter, *D̂* = *D*/*h* for cone (left) and regular square-pyramid (right) tip geometries. Each curve corresponds to a specific tip half-angle, α, as indicated by the different line color and marker style. The curves correspond to a fixed tip–liquid contact angle, θ = 40°. The insets show the simulation snapshots for the corresponding geometry at *D̂* = 10 and α = 14. Detailed simulation plots for other contact angles are available in the Supporting Information (Figure S7).

**Figure 5:**
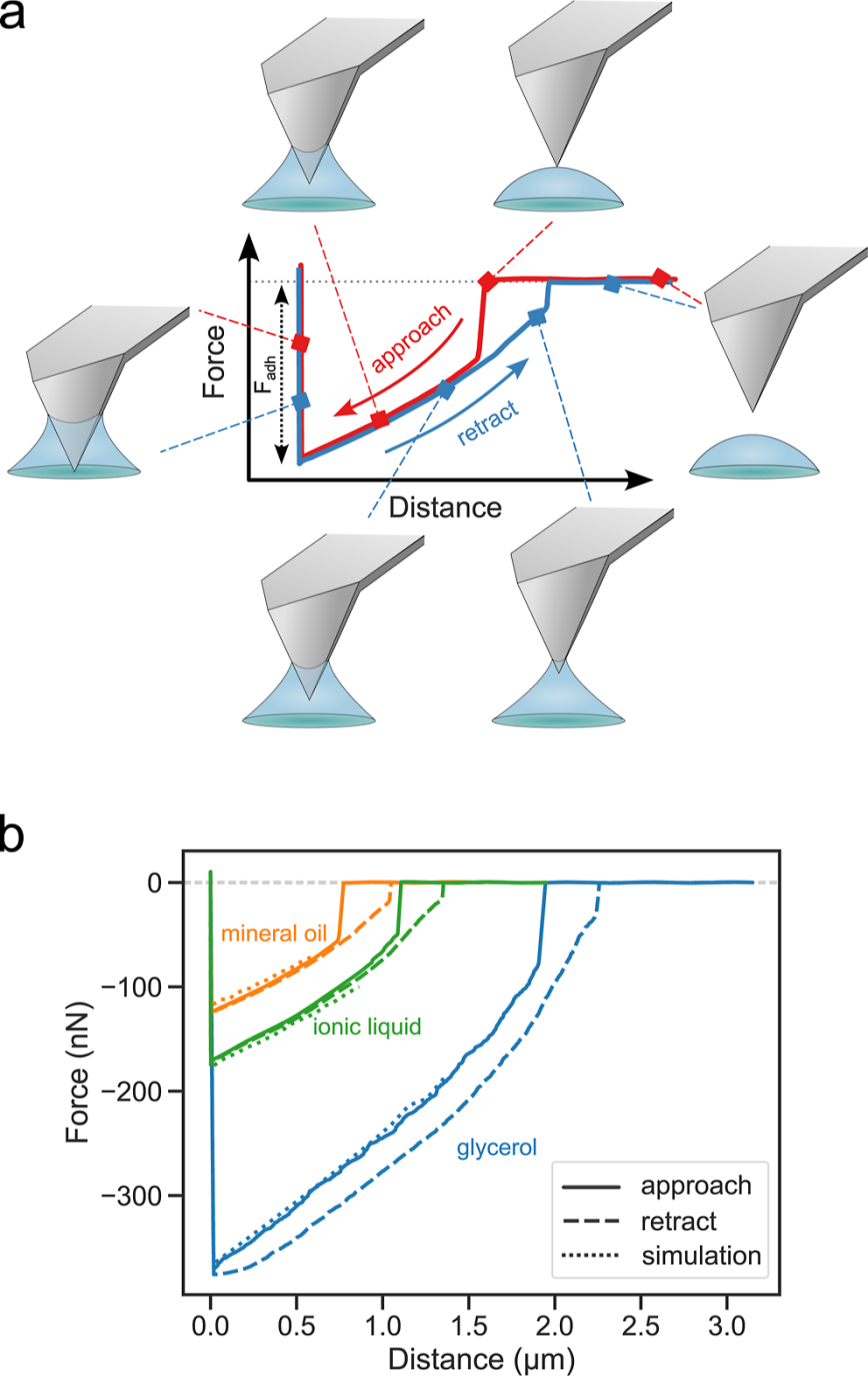
(a) Schematic showing the various stages of contact between a PEG-brush-coated AFM cantilever tip and a liquid droplet during a force measurement. The adhesion force (*F*_adh_) of the drop is measured, as shown. (b) Experimental AFM force curves for mineral oil (orange), ionic liquid (green), and glycerol (blue). The corresponding simulated force curves assuming a pyramidal tip are shown by dotted lines.

**Figure 6:**
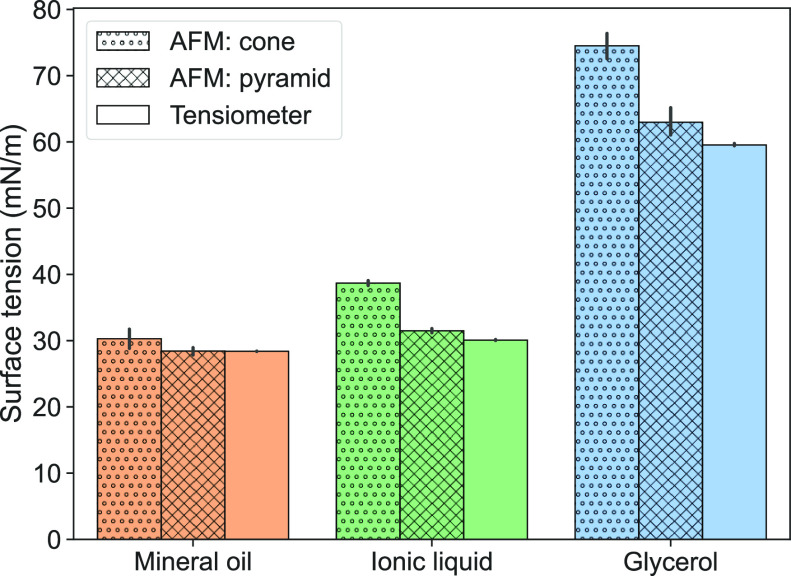
Surface tension of various liquid microdroplets measured using AFM as described in the present work (blue) are compared to bulk liquid measurements using a commercial tensiometer (orange). Detailed data points for individual drops are shown in the Supporting Information (Figure S3).

Overall, for both polar and nonpolar liquids, the AFM method allows for surface tension measurement within a 10% error relative to the expected values. Since the forces measured from an AFM typically have an error on the order of 5–10% due to the uncertainty in spring constant determination, combined with our somewhat simplifying assumptions of the tip shape, the observed deviations are within the range expected. Simulations show a significant impact of tip half-angle, α and tip shape (cone vs pyramid) on the calculated surface tension, due to the sensitive dependence of the tip characteristics on the capillary force (Figure 4). Thus, precise knowledge and modeling of the tip shape are crucial to obtain accurate values of surface tension. Our results suggest that the pyramid-shaped assumption of the tip shape could be a reasonable approximation for surface tension estimation within 10% error. The tip full-angle value provided by the cantilever manufacturer (Bruker) correspond to the pyramidal region rather than the cone region of the tip. Our measurements of the tip full-angle (α_opp_ = 38.1 ± 0.8°) from SEM images, which were then used to obtain the true tip half-angle (α) for surface tension calculations, are within the value provided by the manufacturer (average α_opp_ = 37.5 ± 2°). Thus, one may rely on the tip full-angle value reported in the cantilever specification sheet for further analysis provided that the drop heights are larger than 500 nm. In this case, SEM imaging of the cantilever tip will not be necessary.

The key to make the above measurements possible was the hydrophilic coating of the cantilever tip with a PEG brush. AFM force–distance curves taken at the center of the drop confirm the quality of tip coating, as evidenced by the smooth and almost completely overlapping traces during approach and retract cycles (Figure 5). The low contact angle hysteresis of the coating not only minimizes the accumulation of liquid on the tip over repeated measurements but also ensures that a nearly constant low contact angle is maintained between the tip and the liquid drop. This allows us to simulate our system relatively easily by assuming the ideal scenario of no contact line pinning. The low contact angle between the liquid drop and the tip also ensures a high capillary adhesion force, which minimized any errors in the force measurement due to the contribution of other attractive forces which could influence the net adhesion. For example, the van der Waals adhesion force of the tip with the hard substrate is typically less than 5 nN. This is significantly smaller than its capillary adhesion force with a liquid drop (>100 nN). Thus, van der Waals adhesion can be safely ignored. Alternative hydrophobic coated cantilevers based on the PDMS brush^32^ or fluorosilane did not work as well as a PEG-brush-coated cantilever due to their low capillary adhesion to liquids like glycerol and nonideal AFM force curves resulting from contact line pinning (see the Supporting Information, Figure S1).

Macroscopic dynamic contact angle measurements on flat PEG-brush-coated silicon wafers show that mineral oil and ionic liquid spread quite well on the coated surface, with a contact angle less than 10° (see the Supporting Information, Figure S5). On the other hand, glycerol, which has a relatively high surface tension, showed a receding contact angle of roughly 41° with the same surface. In this work, the surface tension calculations were performed by assuming the coated tip–liquid contact angle (θ) to be similar to those of these experimental measurements. However, our calculation method is sensitive to θ. For example, glycerol was assumed to have a θ of 40°, based on its macroscopic value, which resulted in a surface tension of 62.9 ± 4.6 mN/m following the pyramid approximation (Table 1). On changing θ to 10°, the average surface tension value would, however, drop to 45.1 ± 4.3 mN/m, which would correspond to a 28% measurement error relative to its macroscopic value (see the Supporting Information, Figure S4). In the case of mineral oil and ionic liquid, θ = 10° gives a good prediction of surface tension (<2% error) since their actual contact angles are close to that value (Figure 6). Thus, knowledge of liquid contact angle with a PEG-brush-coated flat surface is essential to improve the estimate of surface tension using AFM data.

Micrometer-sized drops tend to evaporate fast because of the increased vapor pressure due to their highly curved surface. To minimize evaporation, we carried out our measurements in a sealed environment. Since the AFM imaging of a drop and its subsequent force measurement process can take up to 15 min in total, it is important to ensure that the drop does not significantly lose volume during this time. We tracked the drop evaporation by repeated AFM imaging and found less than 5% volume losses, confirming the stability of the drops during measurement (see the Supporting Information, Figure S6). For more volatile liquids such as water, measurements need to be performed under low temperature and saturated vapor conditions. Our preliminary experiments using Cypher AFM (Asylum Research), which has an in-built temperature control of the sample stage, made it possible to make measurements on water microdroplets, which gave a surface tension of ≈67 mN/m (more details are given in the Supporting Information, Figure S2).

In our work, we have reported surface tension measurements of liquid drops in the range of 28–63 mN/m for drop diameters in the range of 5–25 μm. Our method should work for other sample liquids within this range. Beyond this, there may exist certain limitations. (1) Our method relies primarily on the presence of a drop deposited on a flat surface. For the case of small surface tension liquids, the drop would tend to spread out more into a film. In such a case, the normalized drop contact diameter (*D*/*h*) for the liquid film would have a large value. One may obtain a rough surface tension estimate of the thin film from the asymptotic value of the corresponding curve in Figure 4 since they all tend to saturate at high *D*/*h*. The film height, *h*, could be directly measured from the force curve’s “jump-in” point, and together with the adhesion force, *F*_adh_, the surface tension may be estimated. Otherwise, a different substrate with a low surface energy (e.g., PTFE) may be chosen to deposit the drop. Then, the drop would not spread into a film but rather make a finite contact angle with the substrate, similar to our reported measurement conditions. (2) The AFM provides a sufficient resolution to image drops as small as tens of nanometers in diameter and also measure forces in the range of a few nanonewtons by choosing a sufficiently soft cantilever. The challenge for measuring even smaller drops would thus be posed by the tip shape. If the drop diameter is of a similar size as the tip diameter (typically 15–25 nm), a conical or pyramid model will no longer be applicable. For sizes >50 nm, one may choose the conical model. However, here, special attention needs to be paid on the exact tip shape in such small length scales since the shape may deviate quite a lot due to manufacturing defects. (3) As mentioned before, liquid evaporation can have an especially significant effect when measuring small drops. Increased evaporation rate due to the Kelvin effect could result in large changes in the drop volume during the AFM measurement and thus lead to unreliable estimates. (4) The piezo scan range of the AFM limits the maximum drop size that can be measured. Commercial AFMs have a scan range typically on the order of 100 μm in the lateral directions and 10–20 μm in the vertical direction. This may be overcome by instead using the AFM’s motor stage during force measurement and relying on direct optical imaging to obtain the drop size. The drop height also has to be sufficiently small (less than 5–10 μm) so that it makes contact only with the tip’s lateral faces and not with the rectangular cantilever area above. A stiffer cantilever would also be necessary in order to measure the relatively high capillary force of the large drop.

Our method provides an alternative to previously reported AFM-based techniques to measure surface tension, which necessitated fabrication of specially defined tip geometries such as “nanorods”^10^ or “nanoneedles”.^11^ With such special tips, the calculation of surface tension from the measured capillary force is straightforward since the cylindrical shape of the tip keeps the contact perimeter constant. However, fabrication of such special cantilever tips with a uniform geometry is tricky and expensive. Our method uses standard pyramidal tips, which are used widely for general purpose AFM imaging. We coat the tips with a PEG brush. This coating is, however, an easy and inexpensive one-step process, which does not require special equipment or expertise. The relatively longer calculation procedure involved in our method has also been automated with open-sourced Python scripts, making the method easily accessible to a general user.

## Conclusions

We present a method to measure the surface tension of small liquid droplets with a volume in the order of femtoliters. Atomic force microscopy (AFM) was used to image the shape of liquid drops in the tapping mode. In addition, AFM force distance curves were recorded with PEG-brush-coated cantilever tips. Thanks to its low contact angle hysteresis, the PEG coating minimizes the liquid losses or pinning effects of the moving contact line over the tip, resulting in an ideal force response which could be modeled relatively easily. Further, the high surface energy of PEG allows a liquid drop to have a small contact angle with the tip, resulting in an improved measurement sensitivity due to the high capillary force. Simulations of the drop interacting with an approximated tip geometry were performed to calculate the surface tension from the experimentally measured drop adhesion force and drop shape parameters obtained by AFM. Using the pyramidal tip approximation, the resultant surface tension values agree within a 10% error for a range of liquids when compared to macroscopic measurements using a commercial tensiometer.

## Supporting Information Available

Effect of alternative tip coating, measurement on water droplets, raw surface tension data, effect of contact angle, droplet evaporation, macroscopic contact angle images, and simulation plots for a range of contact angle/tip angles (PDF)
Droplet AFM results data (XLSX)
