# Supplementary material for: Method to Measure Surface Tension of Microdroplets Using Standard AFM Cantilever Tips
Source: Langmuir. 2023 Jul 19;39(30):10367–74. doi: 10.1021/acs.langmuir.3c00613 (PMC10399288; doi:10.1021/acs.langmuir.3c00613)
Supplement: Supplementary file 1 — la3c00613_si_001.pdf [file la3c00613_si_001.pdf]

A method to measure surface tension of micro-droplets using standard AFM cantilever tips: Supporting information

Pranav Sudersan,[*,†] Maren Müller,[†] Mohammad Hormozi ,[‡] Shuai Li,[†] Hans-Jürgen Butt,[†] and Michael Kappl[*,†]

†*Max Planck Institute for Polymer Research, Ackermannweg 10, 55128 Mainz, Germany*

‡*Technical University of Darmstadt, Hochschulstraße 8, 64289 Darmstadt, Germany*

E-mail: sudersanp@mpip-mainz.mpg.de; kappl@mpip-mainz.mpg.de

List of Figures

Figure S1 AFM force-distance curves on liquid droplets using PDMS-brush or fluorosilane coated cantilever tips S4

Figure S2 AFM force-distance curves on water drops performed on the JPK NanoWizard 4 (Bruker) attached with a custom made sample stage cooling system (blue) and Cypher AFM (Asylum Research), which has an in-built cooling system (orange). Measurements were performed using AC200TS cantilever tips (9 N/m, 150 kHz) coated with PEG-brush. S6

Figure S3 Detailed measurement data of surface tension of several liquid droplets, calculated using the AFM method as described in the main text. Here, each point represents a measurement done on a unique liquid drop. Calculations based on cone and pyramid approximation of the tip shape are shown separately as circles and crosses respectively. Summarized results of the presented data are reported in Table 1 and Figure 6 of the main text. S7

Figure S4 Surface tension calculation for glycerol are shown by assuming a tip-liquid contact angle of 10°and 40°. The values are reported for both cone and pyramid tip approximations. Here, the tip is coated with PEG-brush. . . . S8

Figure S5 Silicon wafer was coated with PEG-brush as described in the main text. Dynamic liquid contact angles were measured for mineral oil, ionic liquid, glycerol and water by observing the drop sliding over the substrate tilted by 10°. The obtained receding contact angle values here were used as the basis for AFM tip-liquid contact angle assumption for surface tension measurement of each given liquid (reported in Table 1 of the main text). Note that mineral oil and ionic liquid shows a very small contact angle below 10°, where optical measurement of the angle is no longer precise. S9

Figure S6 Glycerol droplet was imaged before and after force measurements (≈10 mins apart) to track its evaporation. The drop volume does not change significantly during this time span, going from 119 fL to 114 fL (4.2% decrease). This corresponds to less than 0.1 mN/m change in calculated surface tension value. Thus, the droplet evaporation rate here can be assumed to not influence the AFM measurements. S10

Figure S7 Simulation curves showing normalized surface tension, $\hat{\gamma} = \gamma h/F_{adh}$ as a function of normalized drop contact diameter, $\hat{D} = D/h$ for cone (left) and regular square pyramid (right) tip geometries for various tip-liquid contact angles (see colour legend) and tip half angle, α. S11

Effect of tip coating

Surface modification of the AFM cantilevers were performed to investigate the effectiveness of alternative coatings that are typically used to reduce the wettability of a surface. Here, we report PDMS-brush coated and fluorosilane coated cantilevers.

RFESPA cantilever tips were coated with PDMS-brush by chemical vapour deposition (CVD) method. 0.1 ml of dichlorodimethylsilane (Sigma-Aldrich) was placed in a sealed 1 Litre chamber together with the plasma treated cantilever tips for 10 minutes. The cantilevers were subsequently rinsed in toluene before AFM measurements. Similarly, fluorinated cantilever tips were also prepared by CVD method, but under vacuum. A small cup containing 0.05 ml of 1H,1H,2H,H-perfluorooctyltrimethoxysilane (Sigma–Aldrich) was placed in a 5 Litre vacuum chamber ($<$ 100 mm Hg) for 10 minutes to fluorinate the tips by the CVD process. The tips were then heated to 150°C for 30 minutes before AFM experiments. Mineral oil and glycerol droplet preparation and subsequent AFM measurement protocol were followed exactly as described in the main text.

Force distance curves (Figure S1) indicate that mineral oil shows high capillary adhesion and little hysteresis when the tip is coated with PDMS-brush, which is a consequence of its low contact angle with the coated tip. Here, the approach and retract curves have a relatively smooth trend during drop contact, similar to our measurements with PEG-brush coated tips. However, for glycerol, the forces curves show a significantly non-ideal trend, with several local pinning events. The measured adhesion force in this case is also quite low, due to the large contact angle that glycerol has with the hydrophobic tip. A similar problem is also seen with the fluorinated tips, where even for mineral oil, the force curves show several pinning events as well as low adhesion. Thus hydrophobic coatings are not a good choice to obtain smooth force-distance curves with high adhesion on liquid droplets, which is necessary to reasonably model the tip-drop contact process for surface tension estimation.

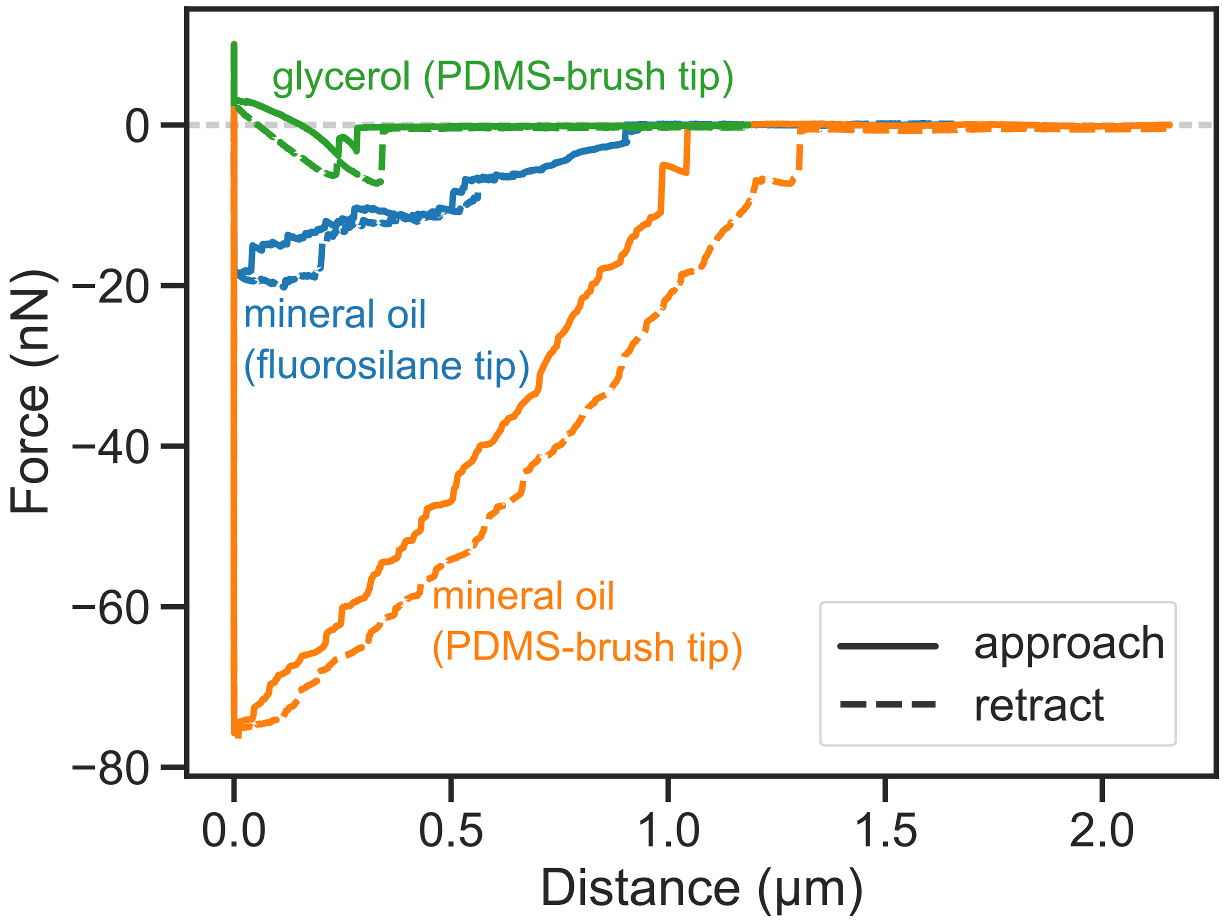

Figure S1: AFM force-distance curves on liquid droplets using PDMS-brush or fluorosilane coated cantilever tips

Measurement on water droplets

Vapour pressure of a liquid depends on the curvature of its air-liquid interface due to the so-called Kelvin effect. This means that liquids like water become extremely volatile when they are in the form of a tiny microscopic droplet. Since the AFM measurement process takes roughly 15 minutes per scan area, it is important that the droplet does not significantly evaporate during such time scales. This may be achieved by proper control of the measurement environment, i.e., under a vapour-saturated and low temperature conditions. Our attempts to perform AFM experiments on water drops using the JPK NanoWizard 4

AFM failed to give reasonable force curves (Figure S2). The laser spot on the cantilever head was too large that it locally heats the water drop during image scanning, even though the measurements were done under sealed saturated vapour conditions at 5°C. Further, our custom made cooling stage introduced unwanted mechanical noise into the system, overall rendering such measurements unfeasible for volatile liquids like water.

On the other hand, our preliminary experiments using Cypher AFM (Asylum Research) gave us a rather smooth and stable force curve for water droplet, showing very little hysteresis (Figure S2). Here, the Cypher AFM had a precisely engineered in-built sample chamber, where the temperature and humidity can be controlled under a sealed environment quite well without introducing noise. More importantly, the laser spot on the cantilever head was focused to a much smaller area in this particular AFM, which significantly minimized droplet evaporation during imaging and force measurements. Based on the measured force curve shown here, using the cone approximation of the tip shape, the surface tension of water was calculated to be $\approx$ 67 mN/m, quite close to the expected macroscopic value of 72 mN/m. Thus our reported method could potentially be extended to other volatile liquid droplets using an appropriate AFM instrument. A proper cooling system and a focused cantilever laser spot are essential for the system to inhibit droplet evaporation during measurements.

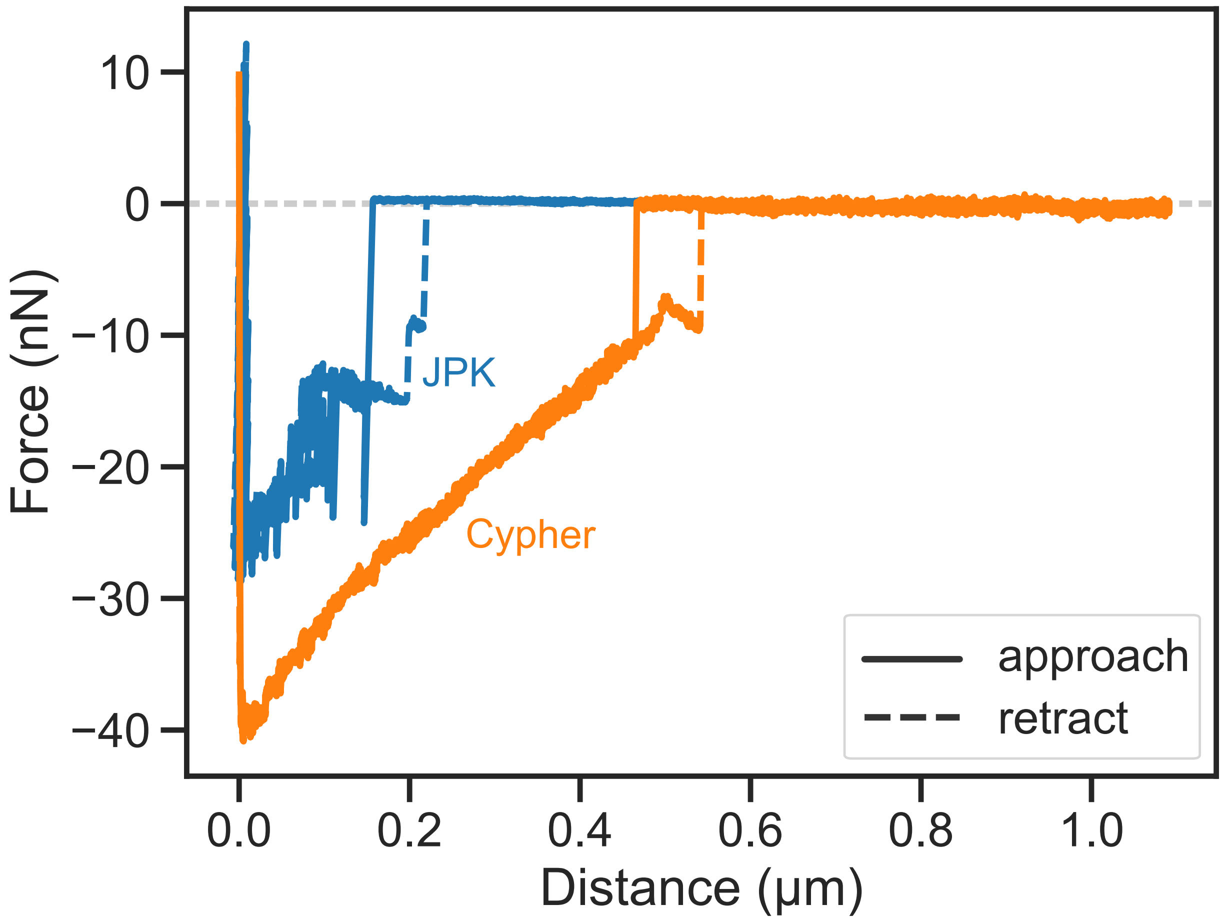

Figure S2: AFM force-distance curves on water drops performed on the JPK NanoWizard 4 (Bruker) attached with a custom made sample stage cooling system (blue) and Cypher AFM (Asylum Research), which has an in-built cooling system (orange). Measurements were performed using AC200TS cantilever tips (9 N/m, 150 kHz) coated with PEG-brush.

Surface tension data

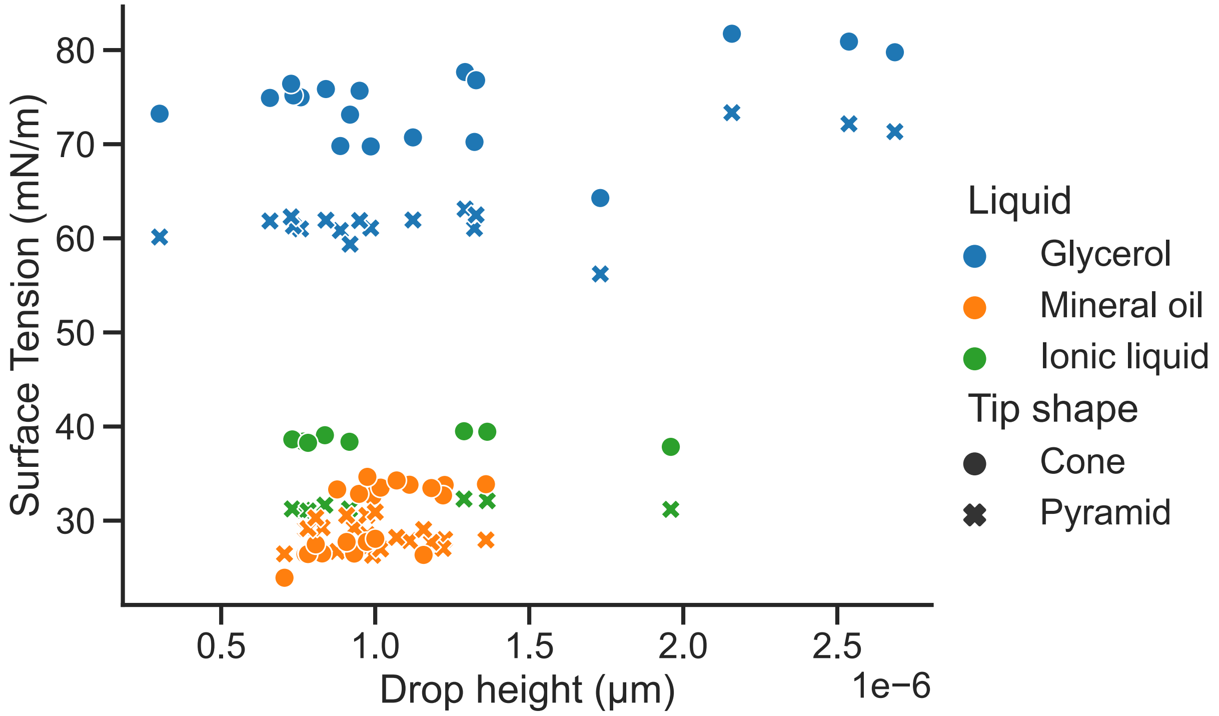

Figure S3: Detailed measurement data of surface tension of several liquid droplets, calculated using the AFM method as described in the main text. Here, each point represents a measurement done on a unique liquid drop. Calculations based on cone and pyramid approximation of the tip shape are shown separately as circles and crosses respectively. Summarized results of the presented data are reported in Table 1 and Figure 6 of the main text.

Glycerol: effect of contact angle

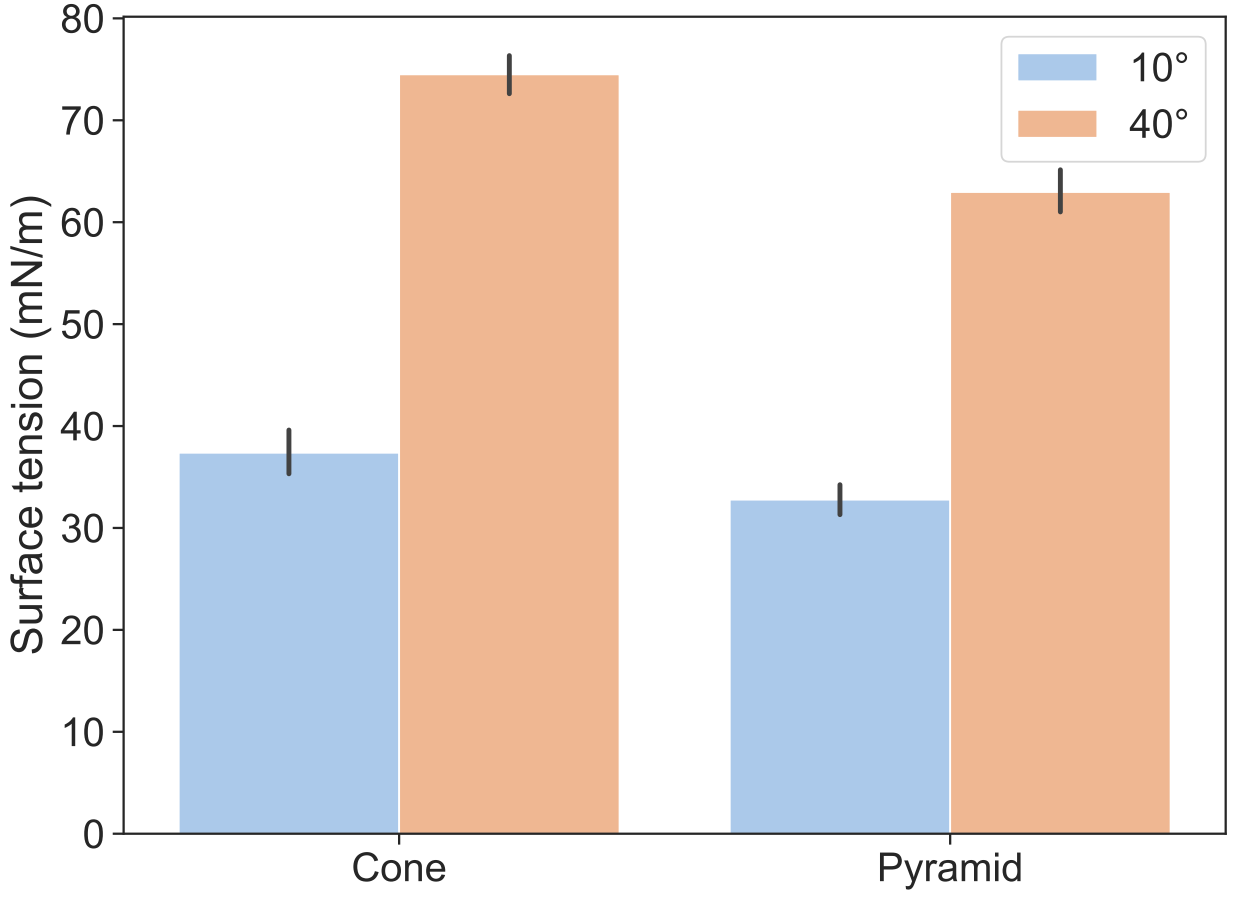

Figure S4: Surface tension calculation for glycerol are shown by assuming a tip-liquid contact angle of 10°and 40°. The values are reported for both cone and pyramid tip approximations. Here, the tip is coated with PEG-brush.

Contact angle measurements

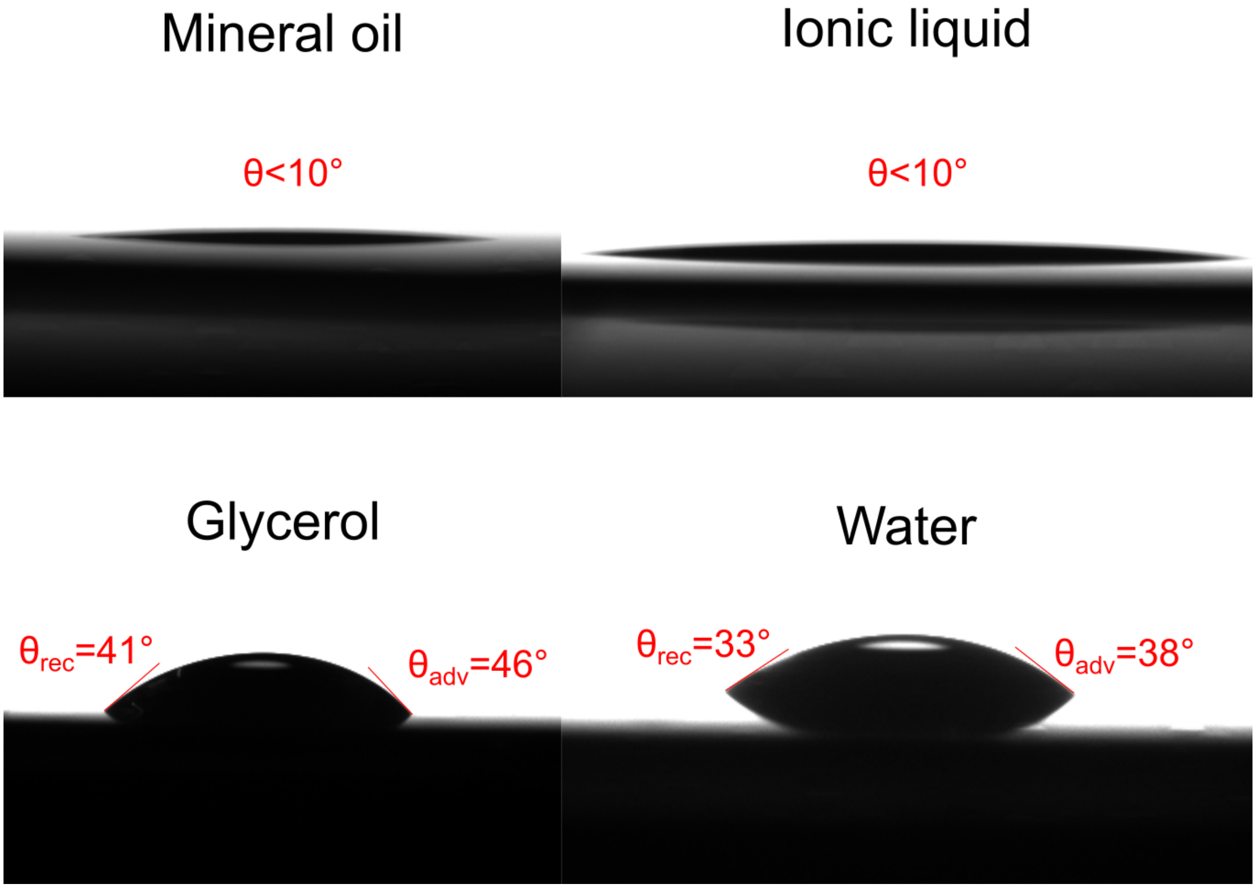

Figure S5: Silicon wafer was coated with PEG-brush as described in the main text. Dynamic liquid contact angles were measured for mineral oil, ionic liquid, glycerol and water by observing the drop sliding over the substrate tilted by 10°. The obtained receding contact angle values here were used as the basis for AFM tip-liquid contact angle assumption for surface tension measurement of each given liquid (reported in Table 1 of the main text). Note that mineral oil and ionic liquid shows a very small contact angle below 10°, where optical measurement of the angle is no longer precise.

Glycerol droplet evaporation

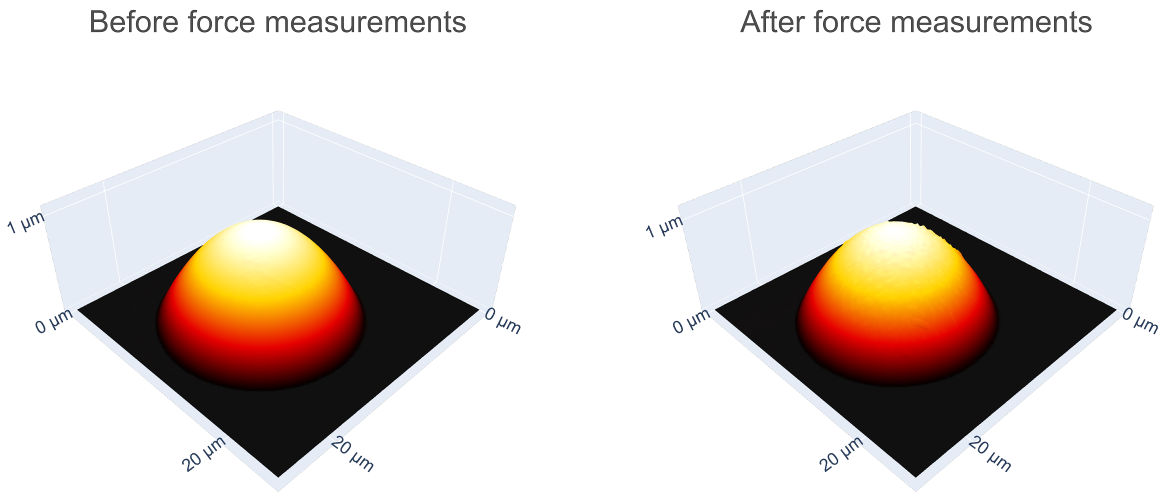

Figure S6: Glycerol droplet was imaged before and after force measurements (≈10 mins apart) to track its evaporation. The drop volume does not change significantly during this time span, going from 119 fL to 114 fL (4.2% decrease). This corresponds to less than 0.1 mN/m change in calculated surface tension value. Thus, the droplet evaporation rate here can be assumed to not influence the AFM measurements.

Simulation plots

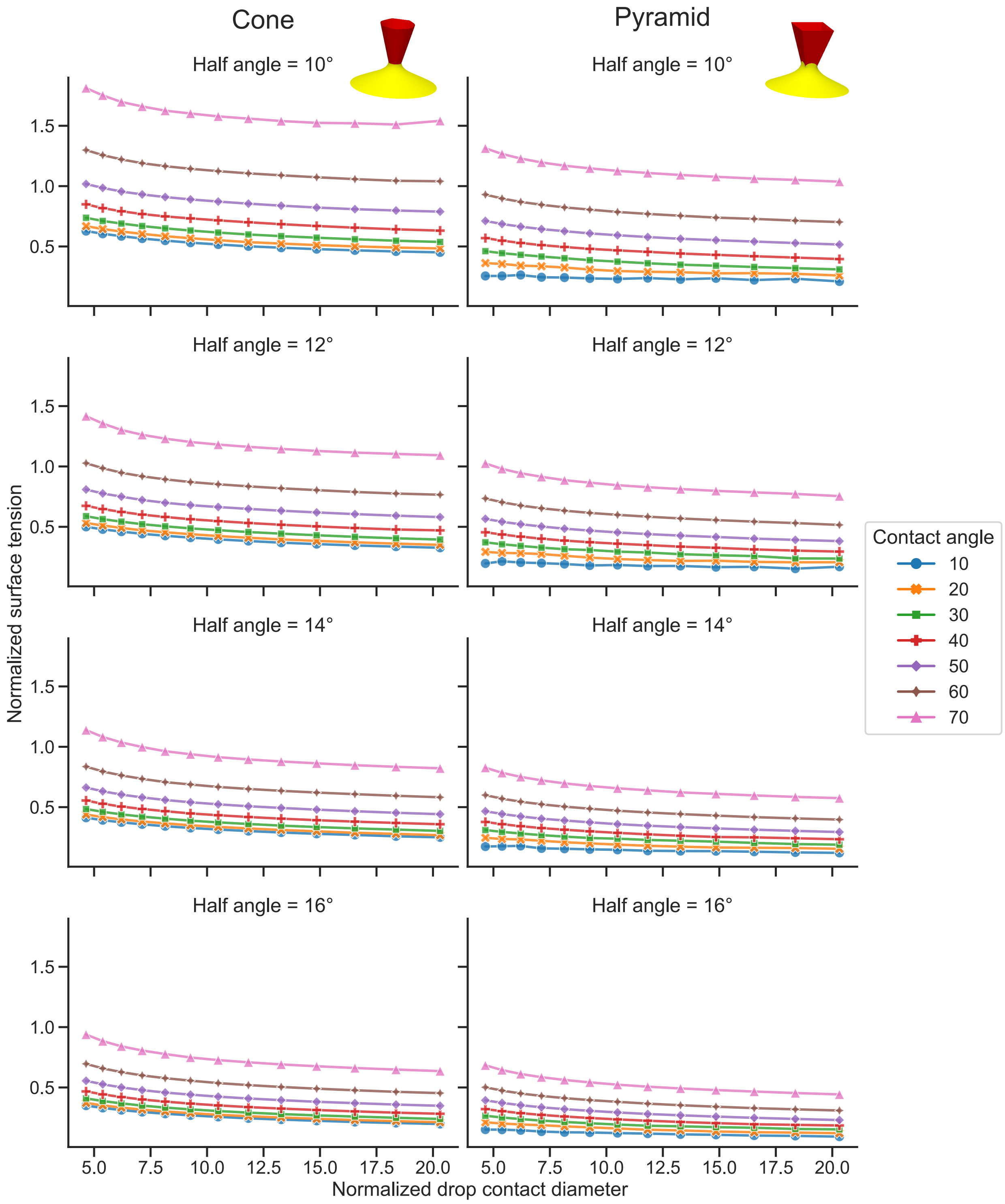

Figure S7: Simulation curves showing normalized surface tension, $\hat{\gamma} = \gamma h/F_{adh}$ as a function of normalized drop contact diameter, $\hat{D} = D/h$ for cone (left) and regular square pyramid (right) tip geometries for various tip-liquid contact angles (see colour legend) and tip half angle, α.
